# Biomechanical comparison of pedicle screw fixation strength in synthetic bones: Effects of screw shape, core/thread profile and cement augmentation

**DOI:** 10.1371/journal.pone.0229328

**Published:** 2020-02-21

**Authors:** Mu-Yi Liu, Tsung-Ting Tsai, Po-Liang Lai, Ming-Kai Hsieh, Lih-Huei Chen, Ching-Lung Tai

**Affiliations:** 1 Ph.D. Program in Biomedical Engineering, Collage of Engineering, Chang Gung University, Taoyuan, Taiwan; 2 Department of Orthopaedic Surgery, Spine Section, Bone and Joint Research Center, Chang Gung Memorial Hospital and Chang Gung University College of Medicine, Taoyuan, Taiwan; 3 Graduate Institute of Biomedical Engineering, Chang Gung University, Taoyuan, Taiwan; University of Extremadura, SPAIN

## Abstract

Pedicle screw loosening resulting from insufficient bone-screw interfacial holding power is not uncommon. The screw shape and thread profile are considered important factors of the screw fixation strength. This work investigated the difference in pullout strength between conical and cylindrical screws with three different thread designs. The effects of the thread profiles on the screw fixation strength of cannulated screws with or without cement augmentation in osteoporotic bone were also evaluated. Commercially available artificial standard L4 vertebrae and low-density polyurethane foam blocks were used as substitutes for healthy vertebrae and osteoporotic bones, respectively. The screw pullout strengths of nine screw systems were investigated (six in each). These systems included the combination of three different screw shapes (solid/cylindrical, solid/conical and cannulated/cylindrical) with three different thread profiles (fine-thread, coarse-thread and dual-core/dual-thread). Solid screws were designed for the cementless screw fixation of vertebrae using the standard samples, whereas cannulated screws were designed for the cemented screw fixation of osteoporotic bone using low-density test blocks. Following specimen preparation, a screw pullout test was conducted using a material test machine, and the maximal screw pullout strength was compared among the groups. This study demonstrated that, in healthy vertebrae, both the conical and dual-core/dual-thread designs can improve pullout strength. A combination of the conical and dual-core/dual-thread designs may achieve optimal postoperative screw stability. However, in osteoporotic bone, the thread profile have little impact on the screw fixation strength when pedicle screws are fixed with cement augmentation. Cement augmentation is the most important factor contributing to screw pullout fixation strength as compared to screw designs.

## Introduction

Pedicle screw fixation has been widely used to treat spinal instability and other spinal disorders, including degenerative spinal diseases, scoliosis, vertebral fractures, spinal infection and metastatic spinal lesions [1–3]. However, screw loosening and pullout resulting from insufficient bone-screw interfacial strength are not uncommon, particularly in patients with osteoporosis [4–7].

Numerous studies have focused on different pedicle screw designs to prevent screw loosening. These designs include screws with an increased outer diameter or length [8], different thread profiles [9–11], a cylindrical or conical core [12–14], expanding screws [15,16] and cannulated screws with polymethylmethacrylate (PMMA) cement augmentation [14,17–19]. All of these screw designs are continuously studied. Among these screw designs, cannulated screws in particular have an efficient alternative and innovative design for preventing osteoporotic incidents when used with cement augmentation [14,17,19–21].

Regarding the design of the pedicle screw thread profile, most pedicle screws in current clinical application are designed with a single-thread profile [22]. A closely spaced, shallow thread design and a larger core-to-outer diameter ratio are typical of cortical screws. In contrast, cancellous screws have widely- spaced, deep threads and a smaller core-to-outer diameter ratio [23]. However, considering pedicle screw instrumentation in spinal surgery, most of the screw anchoring power is contributed from the cortical bone in the pedicle, whereas the contribution is much smaller for the cancellous bone in the vertebral body [24]. It is reasonable to expect that the screw anchoring power can be enhanced if the thread profile accommodated within cortical bone in the pedicle (at the proximal shaft of screw) is designed with fine thread, whereas standard coarse threads (at the tip) gain purchase in cancellous bone. In current study, dual-core/dual-thread (DC/DT) screws are thus designed by equipping a fine thread adjacent to the standard coarse thread on the proximal shaft. Consequently, pedicle screws with a DC/DT design may show an improved screw fixation strength when applied in screw instrumentation surgery. In addition to the thread profile, the screw shape (cylindrical or conical) is considered an influential factor of the screw fixation strength. Abshire [12] reported that conical screws provided higher initial screw holding power than cylindrical screws with identical thread profile design in spinal vertebrae with normal density. However, previous work addressing the combined effects of the screw shape (cylindrical or conical) and thread profile (single thread or dual thread) on screw holding power is lacking.

In osteoporotic bone, cannulated screws with cement fixation have been well described to enhance the screw holding power due to the infiltration of PMMA cement into the trabecular bone [14,17,19–21]. However, to the authors’ knowledge, few available reports have systematically assessed the combined effects of thread profiles and cement augmentation on the screw anchoring power in osteoporotic bone. In current study, the biomechanical performance of the screw shape (cylindrical and conical) of pedicle screws with single fine-thread (SFT), single coarse-thread (SCT), and dual-core/dual-thread (DC/DT) was investigated in normal vertebrae and osteoporotic bones. In addition to the thread profile, the influence of cement augmentation on the screw anchoring power was also examined in osteoporotic bones.

The specific purposes of this study were as follows: 1) to investigate the difference in the pullout strength of solid pedicle screws designed with different thread profiles (SFT, SCT, and DC/DT) and screw shapes (cylindrical and conical) in healthy vertebrae, and 2) to test the influence of the thread profile (SFT, SCT, or DC/DT) of cannulated screws combined with cement augmentation in osteoporotic bones.

## Materials and methods

A total of nine screw designs (6 in each design) were examined in terms of mechanical performance in normal and osteoporotic vertebrae. Standard synthetic L4 vertebrae and low-density test blocks were used as the test objects to represent human normal vertebrae and osteoporotic bones, respectively.

### Synthetic bone samples

Commercially available synthetic L4 vertebrae (Model #3429-4-2, Pacific Research Laboratory, Inc., Vashon Island, WA, USA) were used to model normal spinal vertebrae. The synthetic vertebrae were constructed solid foam cancellous cores with a density of 0.16g/cc, which provided a uniform and consistent morphometry similar to human vertebrae. In addition, a synthetic test block (Model #1522–507) was used to simulate severely osteoporotic bone. These synthetic test blocks were made with open-cell polyurethane foam with a density of 0.12 g/cm^3^, which provided a homogeneous and consistent material with a density in the range of that of human cancellous bone with severe osteoporosis [25].

### Bone screws

For the screw pullout test in the normal vertebrae, six solid screw designs with combinations of two screw shapes (cylindrical and conical) and three thread profiles (SFT, SCT and DC/DT) were employed. For the screw pullout test in the osteoporotic bone (test blocks), three cannulated screw designs with a cylindrical shape and the abovementioned thread profiles were employed.

**Fig 1:**
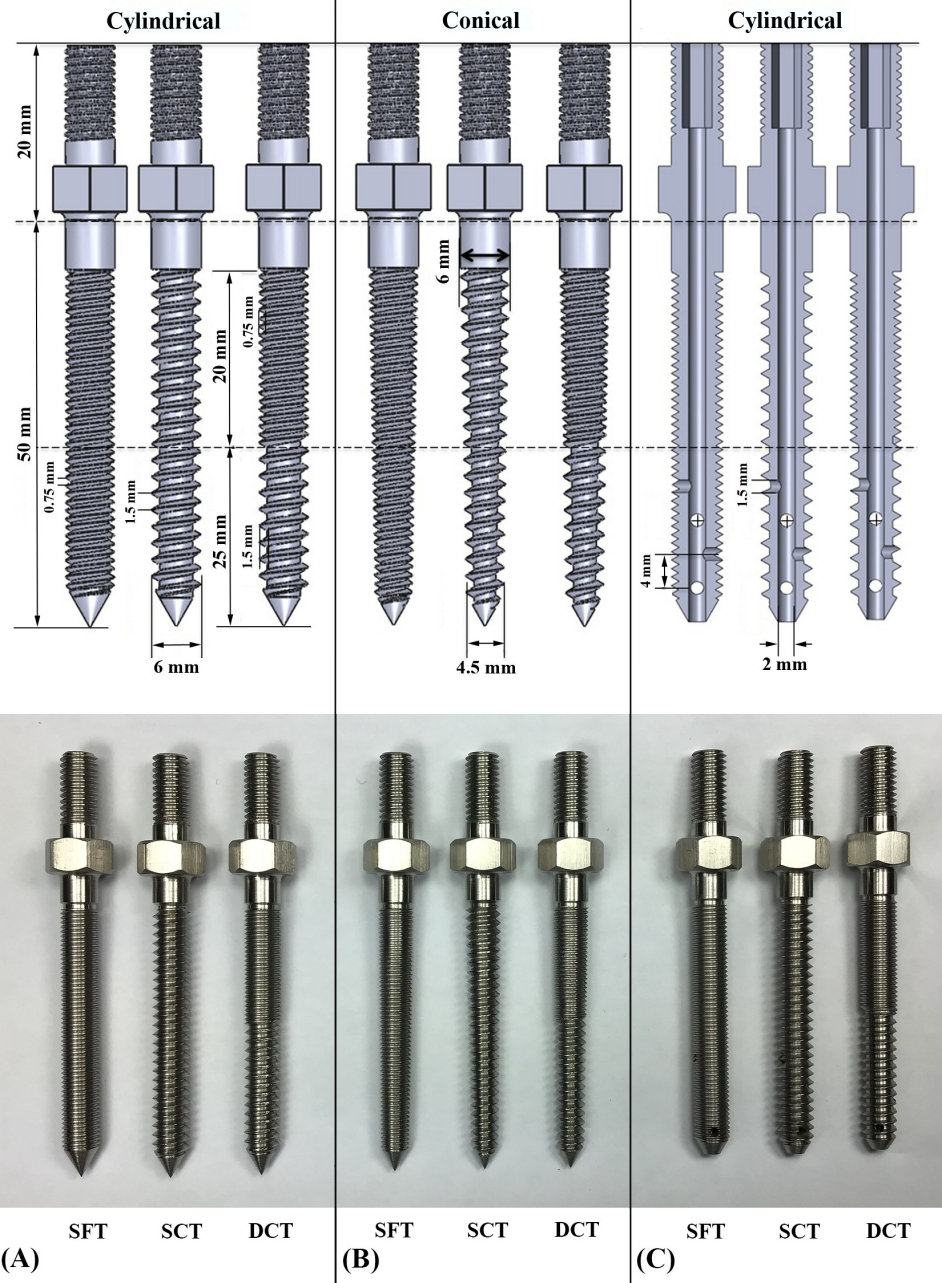
**Schematic drawings (upper) and photographs (bottom) showing nine types of pedicle screws with SFT, SCT, and DC/DT thread profiles.** (A) Solid cylindrical screws; (B) solid conical screws; (C) cannulated cylindrical screws. (⊕ represents a radial hole directed outward from the paper, with dimensions in mm).

All screws have dimensions of 6.0 mm in diameter and 50 mm in length. The specific screw size was chosen to accommodate the geometry of an artificial standard L4 vertebrae into which the screw inserts. The main difference between the conical and cylindrical screws was the taper of the outer geometry from the hub to the screw tip. The cylindrical screws had a constant diameter (6.0 mm) from hub to tip; whereas the conical screws were tapered by 25%, from 6.0 mm at the hub to 4.5 mm at the tip. Both cylindrical and conical screws with the three different thread profiles, i.e., SFT, SCT and DC/DT. For the SFT profile, both the thread pitch and the thread depth were 0.75 mm along the entire length; for the SCT profile, both the thread pitch and the thread depth were 1.5 mm along the entire length; for the DC/DT profile, thread pitch and depth were 0.75 mm in the proximal 20-mm fine-thread region and 1.5 mm in the remaining distal 25-mm coarse-thread region, respectively. Additionally, both the SFT and SCT profiles were designed with a single-start along the entire length, whereas the DC/DT profile was designed with a single-start in the proximal coarse-thread region but two-starts in the fine-thread region (Fig 1). For the cylindrical/cannulated screws (used in the osteoporotic bone), all screw geometries and thread profiles were identical to those of the abovementioned cylindrical/solid screws, except four 2.5-mm radial holes were located at 4-mm increments along the length of the screw starting at the screw tip. Nine groups of pedicle screws with various design parameters are illustrated in Fig 1. The allocation of specimens to experimental groups is listed in Table 1.

**Table 1:** Allocation of the specimens to experimental groups.

Group	Screw Shape	Thread Type	Solid/Cannulated	Augmentation	Synthetic bone used	Specimen Number
1	Cylindrical	SFT	Solid	Cementless	L4 vertebra	6
2	Cylindrical	SCT	Solid	Cementless	L4 vertebra	6
3	Cylindrical	DC/DT	Solid	Cementless	L4 vertebra	6
4	Conical	SFT	Solid	Cementless	L4 vertebra	6
5	Conical	SCT	Solid	Cementless	L4 vertebra	6
6	Conical	DC/DT	Solid	Cementless	L4 vertebra	6
7	Cylindrical	SFT	Cannulated	Cemented	Test block	6
8	Cylindrical	SCT	Cannulated	Cemented	Test block	6
9	Cylindrical	DC/DT	Cannulated	Cemented	Test block	6

### Specimen preparation

#### Standard L4 vertebral specimens (instrumented with solid screws)

A total of 36 standard L4 vertebral specimens were divided into six test groups (6 specimens per group) based on different combinations of two screw shapes and three thread profiles. The method for the insertion of solid screws within standard L4 vertebral specimens was identical to that of our previous study [26]. For all standard vertebral specimens, a 3-mm pilot hole was created along the pedicle and parallel to the superior vertebral endplate with use of a drilling machine [26]. Following creation of the pilot holes, the pedicle screws were inserted along the pilot holes, and a consistent depth gauge was used to ensure that all screws were inserted to the same depth. The accuracy of screw placement was checked using radiological examinations.

#### Osteoporotic test blocks (instrumented with cannulated screws)

The method for the insertion of cannulated screws with cement augmentation was identical to that described in our previous study [17]. Osteobond bone cement (Zimmer, Warsaw, IN) was used for screw augmentation. PMMA cement was injected into the test block after screw insertion. In the beginning, a pilot hole was created using a 3-mm drill bit, and then a cannulated screw was inserted into the test block through the prepared pilot hole. Following screw insertion, a custom-made cement injector capable of exerting pressure on the cement was used to introduce bone cement into the cannulated screws. For all specimens, a total of 3 ml of cement was injected into the screw. Following specimen preparation, radiological examinations of the inserted screws were conducted to check the implanted screw depths.

### Screw pullout test

#### Standard L4 vertebral specimens (instrumented with solid screws)

**Fig 2:**
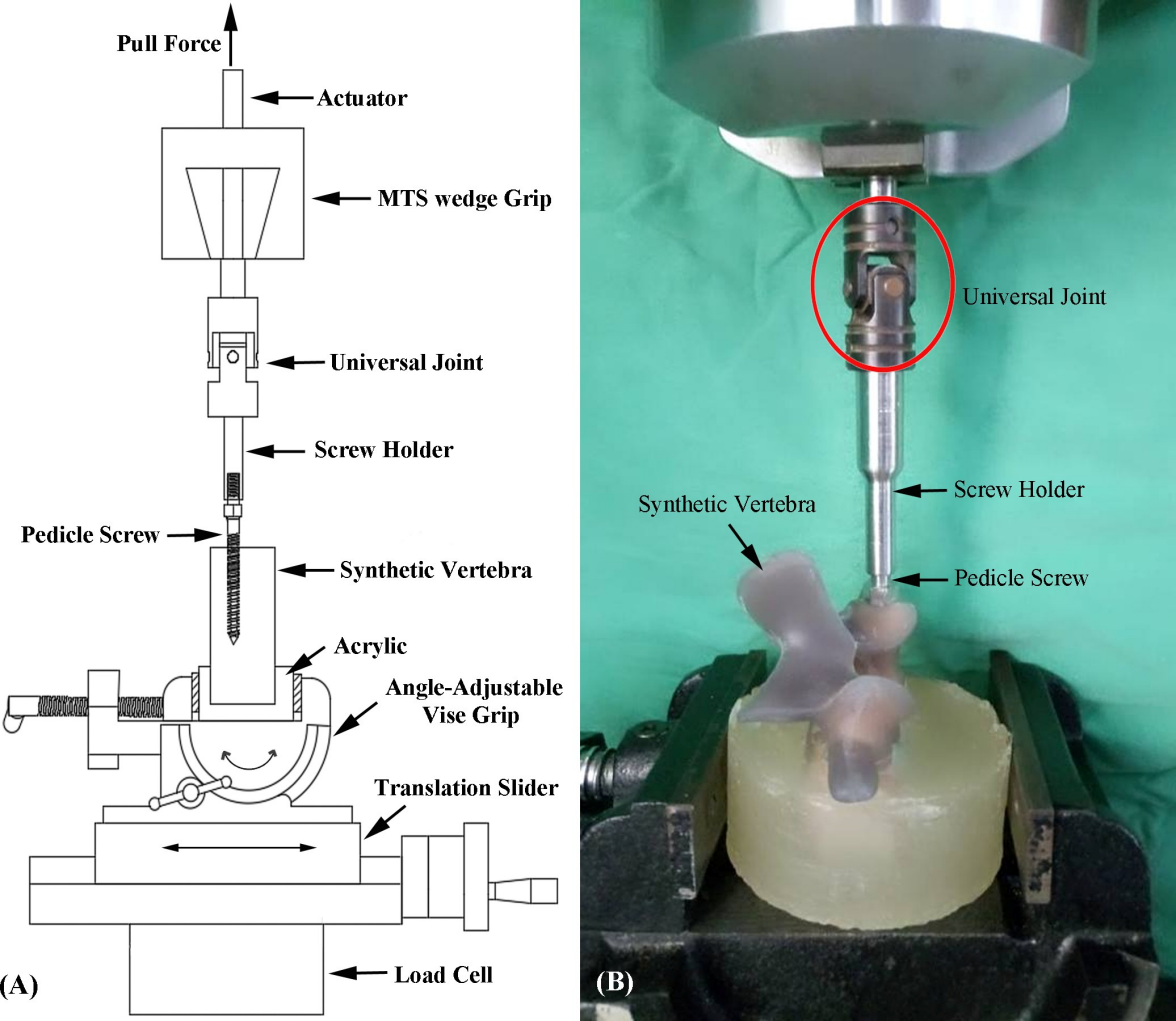
(A) Schematic drawing and (B) photograph showing the screw pullout test in a standard L4 vertebral specimen. Each prepared specimen was secured to a custom-made grip mounted on the platform of the testing machine. The pedicle screw was secured to a cylindrical adapter with universal joint, and the cylindrical adapter was fixed to the upper wedge grip of the testing machine.

Following screw insertion, the instrumented vertebral specimens were embedded in acrylate resin (#20–3568; Buehler, Lake Bluff, IL, USA) to allow clamping for the pullout test. The posterior elements of the vertebra were not embedded. Each prepared specimen was then secured to a custom-made grip mounted on the platform of the testing machine (Bionix 858; MTS Systems Corp., MN, USA). The pedicle screw was secured to a 10-mm cylindrical adapter with universal joint, and the cylindrical adapter was fixed to the upper wedge grip of the testing machine. Following the experimental setup of the specimen, a length-controlled pullout force with a constant crosshead rate of 5 mm/min was applied to the screw head [27]. During the pullout test, the relation between the applied force and displacement was simultaneously recorded in 0.05-mm increments until failure. The experimental setup of the screw pullout test of the standard L4 vertebral specimens is shown in Fig 2.

#### Osteoporotic test blocks (instrumented with cannulated screws)

Each prepared specimen was tested for failure on axial pullout using an Instron testing machine (model 5544, Instron Inc., Canton, MA, USA). The prepared test block was placed on an upper custom-made fixture capable of self-alignment to ensure coaxial alignment of pedicle screw with the pullout ram. The pedicle screw was connected to a cylindrical rod secured to the testing machine. After the specimens were mounted, the testing conditions were identical to that used in our previous study [17]. All the setup steps described above were to ensure that the pedicle screws and the pullout force were directed along the same axis.

### Statistical analysis

The magnitudes of the ultimate pullout force were statistically compared. Statistical software (SPSS for Windows version 12.0, SPSS Inc., Chicago, IL) was used to evaluate the effects of the screw design (conical or cylindrical) and thread profiles (SFT, SCT, or DC/DT) on the stability of spinal fixation. An ANOVA test with post hoc analyses was performed to evaluate the differences between groups. Differences were considered significant at *p* < 0.05.

## Results

### The effect of screw shape and thread profile in healthy vertebrae

**Fig 3:**
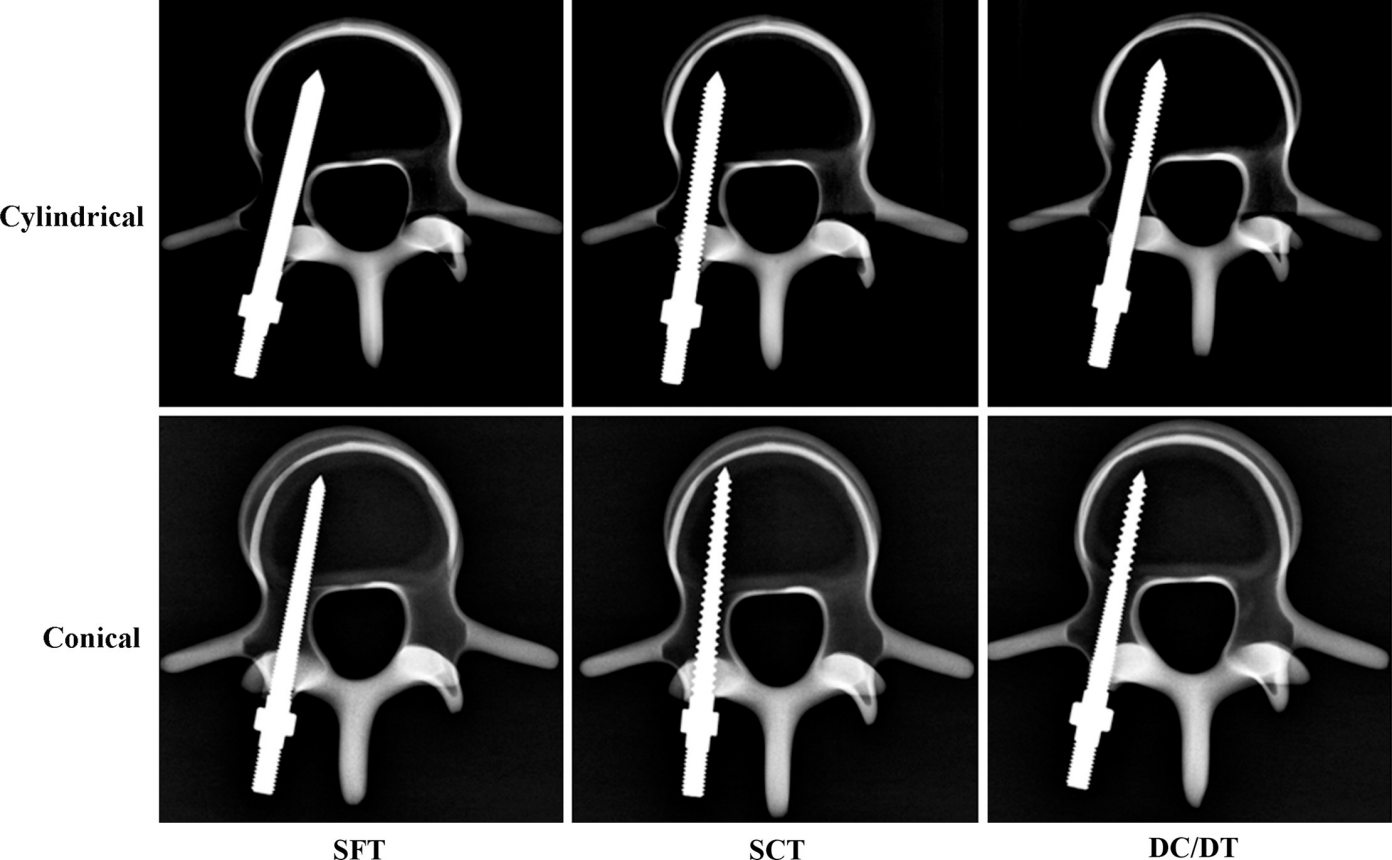
Radiological images showing synthetic vertebrae and the inserted solid screws. The radiological examinations indicated that the proximal fine thread in the DC/DT profile is adequately placed within the region of the pedicle cortical bone, whereas the distal coarse threads gained purchase in the cancellous bone within the vertebral body.

Radiological images of the solid conical and cylindrical pedicle screws with the SFT, SCT and DC/DT profiles inserted into the standard vertebrae are shown in Fig 3. The radiological examinations indicated that the proximal fine thread in the DC/DT profile is adequately placed within the region of pedicle cortical bone, whereas the distal coarse threads gained purchased in the cancellous bone within the vertebral body.

**Fig 4:**
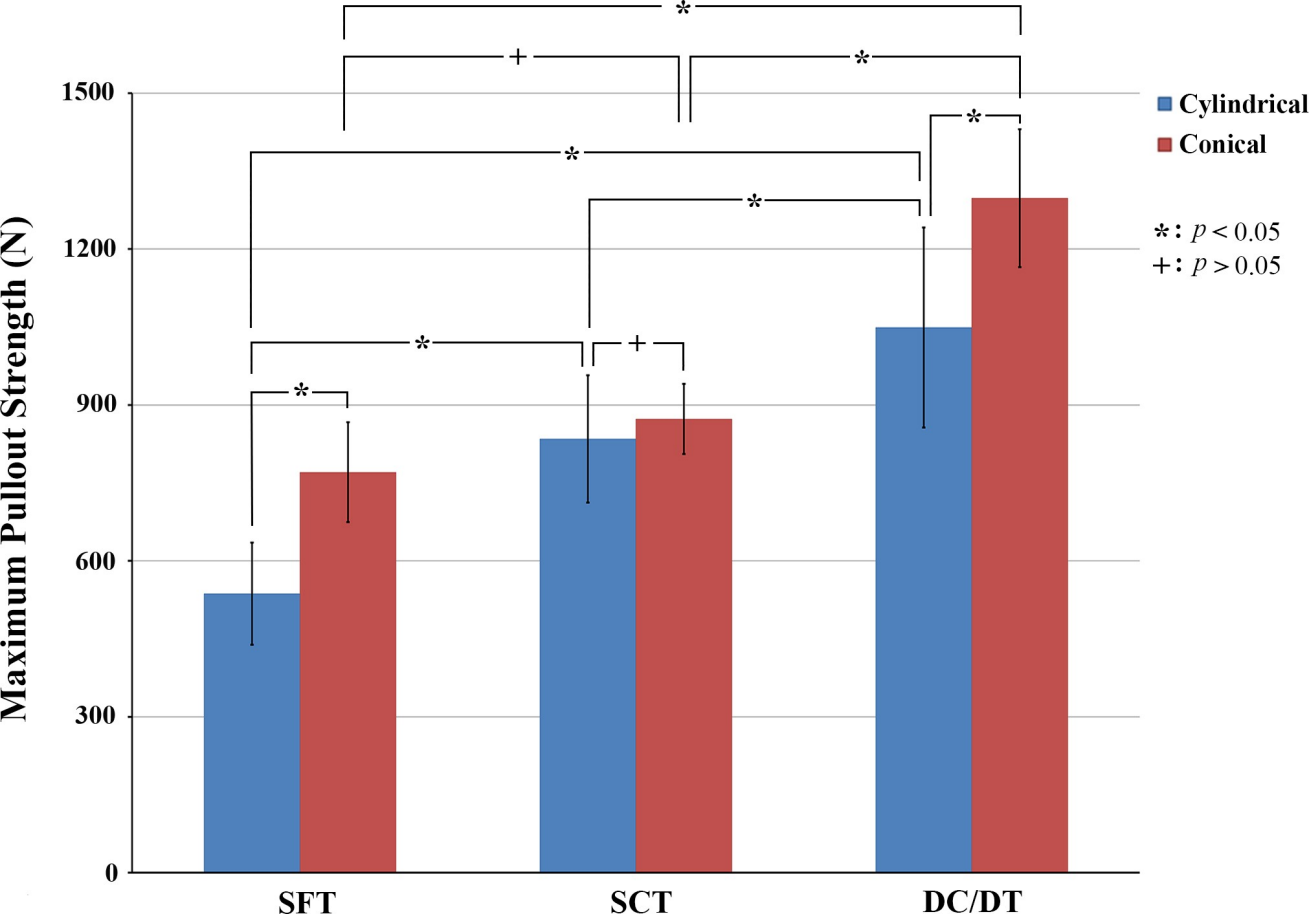
Mean maximal pullout strengths in six groups of synthetic vertebrae instrumented with solid screws. Regardless of the screw outer geometry (conical or cylindrical), solid screws with DC/DT thread profiles exhibited significantly higher pullout strengths than those with SFT or SCT profile.

The average maximal pullout strength of the inserted conical and cylindrical pedicle screws with different thread profiles is shown in Fig 4. The average maximal pullout strengths of cylindrical screws with the SFT, SCT, and DC/DT profiles were 537.06 ± 98.46 N, 834.78 ± 122.43 N, and 1049.11 ± 192.65 N, respectively. The average maximal pullout strengths of conical screws with the SFT, SCT, and DC/DT profiles were 770.46 ± 96.08 N, 873.17 ± 67.45 N, and 1297.78 ± 132.63 N, respectively. Regardless of the screw outer geometry (conical or cylindrical), solid screws with a DC/DT thread profile exhibited a significantly higher pullout strength than those with the SFT or SCT profile. For the SFT and DC/DT profiles, however, conical screws exhibited a higher pullout strength than cylindrical screws (*p* < 0.05).

For the conical screws, the DC/DT profile provided 49% and 68% increases (*p* < 0.05) in the pullout strength compared to the SCT and SFT profiles, respectively. In contrast, for the cylindrical screws, the DC/DT profile provided 26% and 95% increases in the pullout strength compared to the SCT and SFT profiles, respectively (*p* < 0.05). Among the six solid screws, the conical screw with the DC/DT profile presented the highest pullout strength (*p* < 0.05).

### The effect of thread profile and cement augmentation in osteoporotic bone

**Fig 5:**
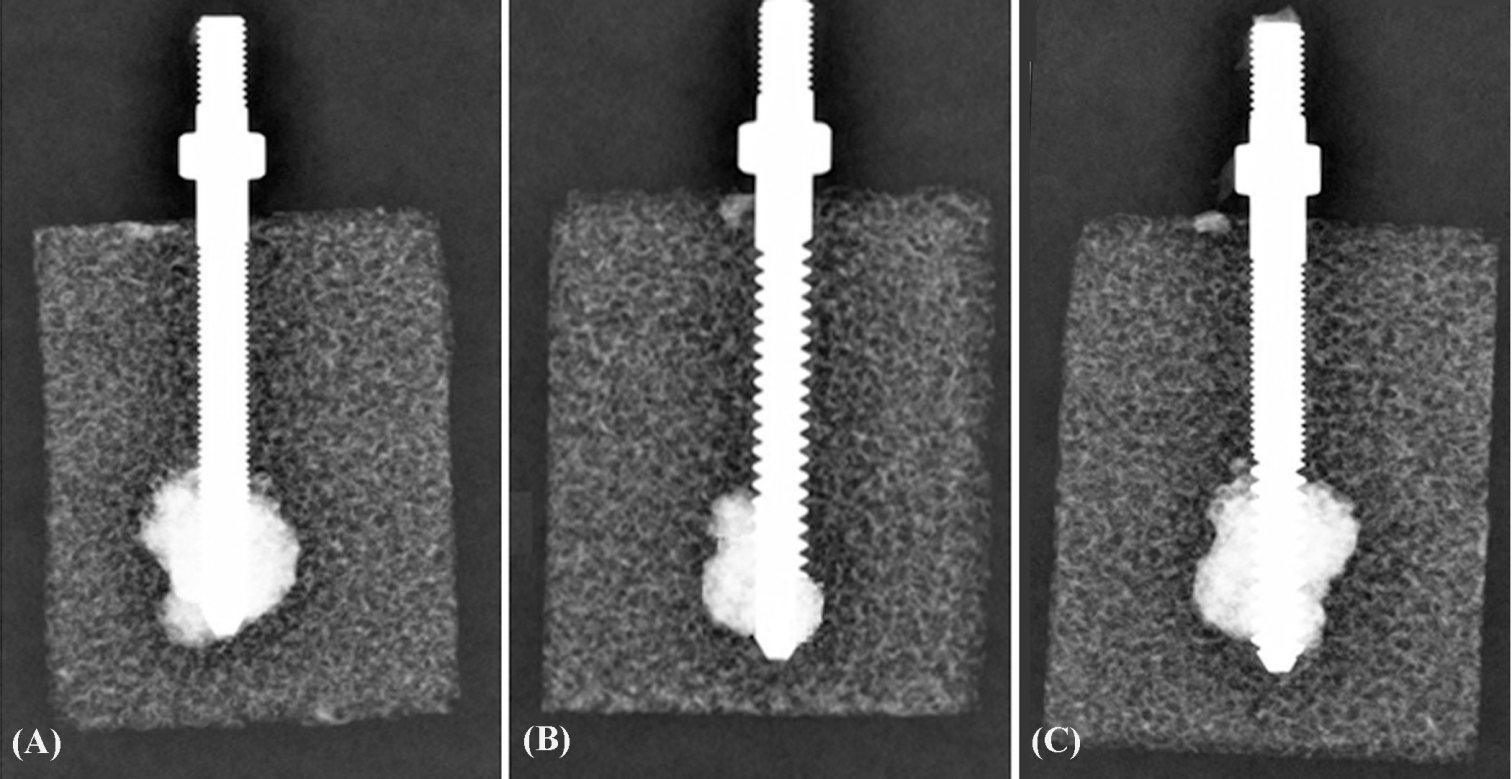
Radiological images showing test blocks and the inserted cannulated screws following cement injection. (A) SFT; (B) SCT; and (C) DC/DT. The radiological examinations indicated that the cement was exuded from the radial holes in the flow path.

**Fig 6:**
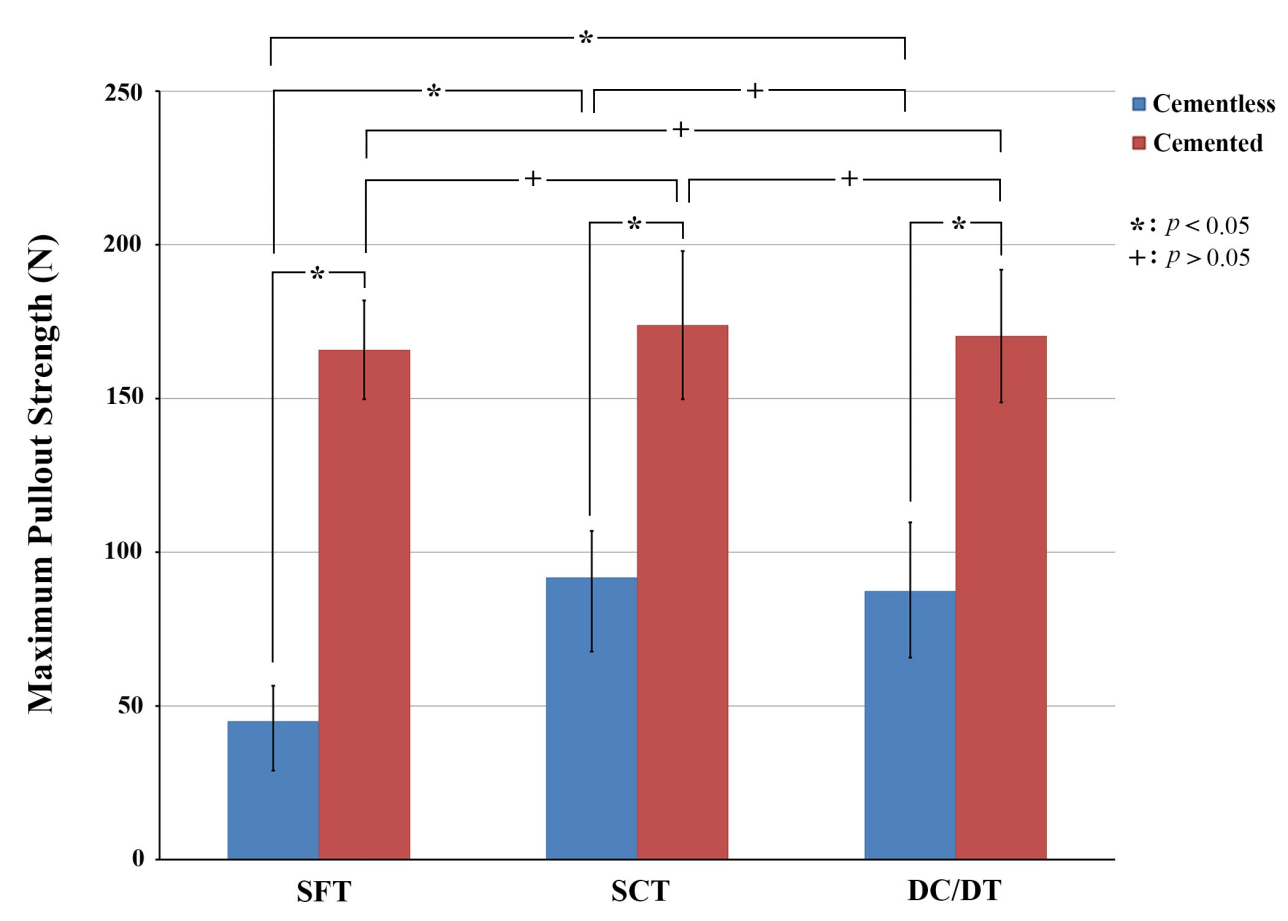
Mean maximal pullout strengths in three groups of test blocks instrumented with cannulated cylindrical screws with and without cement augmentation. The strength was significantly higher with than without cement augmentation (*p* < 0.05). However, no significant difference was found among cemented screws with the SFT, SCT, and DC/DT thread profiles (*p* > 0.05), suggesting that the thread profile has little impact on the screw fixation strength of pedicle screws fixed with cement in osteoporotic bone.

Radiological images of cannulated screws with the SFT, SCT and DC/DT profiles inserted into test blocks are shown in Fig 5. The radiological examinations indicated that the cement was exuded from the radial holes in the flow path. The mean maximum pullout strength for various screw designs with or without cement augmentation is shown in Fig 6. For the SFT, SCT and DC/DT profiles, the average maximal pullout strength of pedicle screws with cement augmentation was 165.85 ± 16.07 N, 173.88 ± 24.06 N, and 170.36 ± 21.56 N, respectively. The strength was significantly higher with than without cement augmentation (*p* < 0.05). The average maximal pullout strengths of pedicle screws without cement augmentation were 45.05 ± 11.56 N, 91.74 ± 15.18 N, and 87.36 ± 22.36 N, respectively. However, no significant difference was found among cemented screws with the SFT, SCT, and DC/DT thread profiles (*p* > 0.05), suggesting that the thread profile has little impact on the screw fixation strength of pedicle screws fixed with cement in osteoporotic bone.

## Discussion

Transpedicular screw instrumentation has been regarded as a successful surgical technique in the treatment of spinal disorders because it ensures a three-dimensional structure over the vertebral motion level, achieving a stiff stabilization. However, screw loosening may occur before spinal fusion takes place in the fixed segments and lead to surgical failure. A previous report indicated a higher failure rate of up to 11% for long-term pedicle screw instrumentation [28]. Among various types of failure, screw pullout is one of the most common clinical complications [29, 30].

Numerous reports have indicated that pedicle screw anchoring power is associated with the screw shape, thread profile, screw pitch and screw diameter [8–14]. Our results indicate that in healthy vertebrae, pedicle screws with a conical shape and DC/DT design have the most robust fixation strength compared to five other screw designs, i.e., the highest pullout strength was found in the combination of conical shape and DC/DT design. These results are consistent with those of previous reports focusing on relative subjects [12, 22]. Traditional pedicle screws are designed with a cylindrical shape and single-thread profile. Conical screws have been reported to possess superior anchoring power compared to cylindrical screws [12]. The higher anchoring power of conical screws is attributed to a better adaptation to the pedicle anatomy, which allows conical screws to achieve increased embedding bone within the screw thread at the cortical/cancellous interface. In addition to the geometric matching of conical screws with the pedicle, the contact of conical screws with the surrounding bone progressively tightens with each turn of the screw during insertion, which enhances the screw fixation strength through compression force [12].

**Fig 7:**
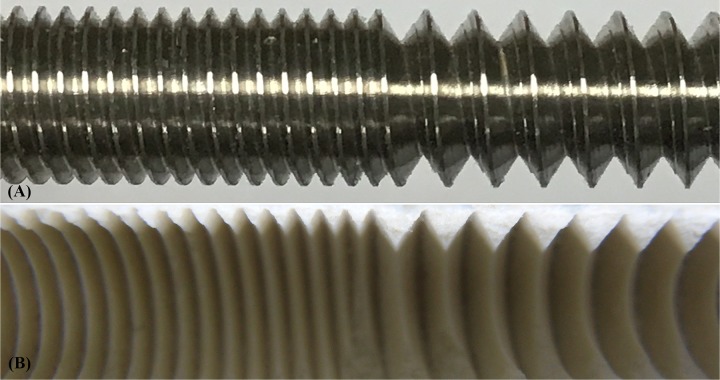
Photographs showing the (A) actual DC/DT screw; and (B) inner thread profile of the test block following insertion and removal of the DC/DT screw. The integral thread profile showed no damage in the test block, demonstrating no interference at the bone-screw interface during screw insertion and removal.

Pedicle screws continue to be studied with different design parameters to improve their anchoring power. In terms of the dual-threaded screw, Brasiliense et al. [22] used human vertebrae and PU foam blocks to compare the screw insertion torque and ultimate pullout force between conventional and dual-threaded screws. Their results revealed a 183% increase in screw insertion torque for the dual-threaded screws than the conventional screws, and the pullout strength of the standard screws was 93% that of the dual-threaded screws. In current study, the DC/DT screws consisted of two different thread profiles (fine and coarse). In the configuration of such a screw design, the problem of interference between the threads and bone during screw insertion needs to be considered [31]. From a mechanical engineering perspective, in the design of a common single-threaded screw, the “pitch” is the distance between screw grooves, whereas the “lead” is the linear distance the screw travels per screw revolution [32]. In such cases, the lead should equal the pitch of the screw. For dual-threaded screws, however, the lead in the coarse-thread region would be twice that in the fine-thread region because the pitch of the coarse threads is twice that of the fine threads. The unequal linear travel between the fine- and coarse-thread regions in a single screw would raise the problem of damage to the bone matrix caused during screw insertion. To solve this problem, a two-start design for the fine threads is required to achieve consistent linear travel for the fine- and coarse-thread regions per screw revolution, which eliminates bone-thread interfacial interference, and prevents damage to the bone matrix. Based on the abovementioned consideration, the fine-thread region of the DC/DT screws was manufactured with a two-start design, whereas the coarse-thread region was manufactured with a one-start design [31]. To confirm that insertion of the DC/DT screw would not raise concerns about bone-screw interfacial interference, an additional test to examine the thread profile within the test block following careful insertion and removal of the DC/DT screw was conducted in the present study. In this test, a DC/DT screw was inserted into the test block (Model: #1522–03, Pacific Research Laboratory Inc., Vashon Island, WA, USA) and was then removed with care. Fig 7 shows the thread profile in the test block following insertion and removal of the DC/DT screw. The integral thread profile showed no damage in the test block, demonstrating no interference at the bone-screw interface during screw insertion and removal.

Numerous biomechanical research studies using artificial standard vertebrae (with integral geometry of a single vertebra) [26,33], and polyurethane (PU) foam blocks [20,33,34] to mimic healthy vertebrae and osteoporotic cancellous bone, respectively, have suggested that these synthetic bones are good alternative choices for *in vitro* experiments. In our recent study [33], artificial standard L4 vertebrae mimicking healthy vertebrae were used to compare the screw anchoring power between intact and broken pedicles. The results revealed that the absence of pedicle would lead to a significant loss of screw anchoring power. Once the pedicle is broken, a revision with a larger or longer-diameter screw is suggested. Ramaswamy et al. [20] used polyurethane (PU) foam blocks with densities of 0.32, 0.24 and 0.16 g/cm^3^ to compare the screw fixation stabilities of various perforated screws. They declared that test blocks with densities of 0.32, 0.24 and 0.16 g/cm^3^ can be used to mimic normal, osteopenic and osteoporotic bones, respectively. They concluded that screw fixation strength had significant correlations with bone density and thread design. Hashemi et al. [34] used PU blocks with densities of 0.32 and 0.16 g/cm^3^ to mimic the porosity of normal and osteoporotic bones, respectively, to compare the pullout strength and insertion torque of pedicle screws with or without cement augmentation. Their report indicated a significantly positive correlation between screw pullout strength and insertion torque. Additionally, Patel et al. [35] conducted mechanical tests on polyurethane (PU) foam blocks with densities of 0.09, 0.16 and 0.32 g/cm^3^ to investigate if these commercial products are appropriate to simulate cadaveric osteoporotic cancellous bone. Their results suggested that PU foam blocks with densities of 0.32, 0.16 and 0.09 g/cm^3^ are suitable to be used as substitutes for normal, osteoporotic and very low density osteoporotic cancellous bone, respectively.

In the present study, only one amount (3 ml) of PMMA cement was tested, adapted from the literature [14,17,36]. Although a total of 3 ml cement was injected into the cannulated screw, the accurate amount of cement infiltration into the test blocks was less than 3 ml because the space in the screw’s central hole would be occupied with a portion of the injected cement. Liu et al. [36] compared the screw pullout strengths of pedicle screws augmented with varying volumes of PMMA (0, 1, 2, 3, 4, and 5 ml) using different densities of polyurethane foam blocks. Their results suggested that PMMA amounts of 3 and 4 ml were preferred in osteoporotic and severely osteoporotic blocks, respectively. In our previous studies [14], test blocks were used to investigate the differences in pullout strength between two different cement augmentation techniques. A total amount of 3 ml of PMMA bone cement was used for cement augmentation in both techniques. The results indicated that “solid screws with retrograde cement prefilling offer improved initial fixation strength when compared to that of cannulated screws with cement injection” [14].

Human cancellous bone has a wide variety of density. It is generally reported that cancellous bone has densities ranging from 0.09 to 1.25 g/cm^3^ [25]. In the present study, PU foam test blocks with a density of 0.12 g/cm^3^ were chosen to simulate severely osteoporotic cancellous bone for the ease of cement injection. We believe that our experimental design with a combination of 0.12 g/cm^3^ polyurethane foam and 3 ml of PMMA volume provides a uniform platform for comparison of the fixation strengths of various pedicle screw designs.

Screw pullout tests in *in vitro* experiments have been extensively used to evaluate the efficacy of new screw designs that promise to enhance the screw stability [8–10, 13–18,33,34,36]. For the screw pullout test, a gradual axial force is applied to a screw that has been inserted into bone, and the maximum force required to loosen the screw is measured. This measured maximum force is then defined as the screw pullout strength. Although axial failure of a screw does not occur often in clinical conditions, the availability and reliability of the screw pullout test render it the most effective way to evaluate a screw’s fixation stability following screw insertion [33, 37]. In the present study, although only axial screw pullout was used to evaluate screw anchoring power without consideration of complex multidirectional loading, the axial screw pullout test is considered an efficient method for comparison of the relative screw anchoring power following screw implantation.

Our study has a number of limitations. First, synthetic composite bone specimens were used as substitutes for human bones. In particular, in the evaluation of cannulated screws inserted in osteoporotic bone, low-density test blocks were chosen to simulate osteoporotic bone rather than synthetic vertebrae because synthetic osteoporotic vertebrae cannot be accessed. This may have had an impact on the bone-screw interfacial strength because the complex geometry of actual vertebrae was not considered. However, the test blocks were made of uniform polyurethane foam, which reduces the influences of the variability of properties and the morphometry of cadaveric bones and provides an effective platform for comparison of the mechanical characteristics of various screw designs in a synthetic osteoporotic model [14,15,17]. Second, only one model of synthetic bone was used to simulate healthy and osteoporotic vertebrae. Bone density plays an important role in determining pedicle screw anchoring power. Explorations into the effect of bone density on screw anchoring strength deserve further study. Third, only one pilot hole size (3-mm in diameter) was tested for all cases. The pilot hole size may enormously affect the screw pullout strength. The combined effects of pilot hole size, screw shape and thread profile should be considered. Fourth, in the cannulated screw pullout test, only one volume (3 ml) of PMMA bone cement was examined using the low-density test blocks [14,17]. The amount of bone cement injected within cancellous bone has great impact in determining the screw anchoring power. Further investigations with various cement volumes might be necessary. Last, only one loading mode (screw pullout test using synthetic bone) was conducted without consideration of other physiological loading modes. In daily activity, the pedicle screws *in vivo* are subjected to complex multiaxial loadings; subsequent investigations into the influence of other loading modes, such as lateral bending and dynamic fatigue, should be performed in the future. Nevertheless, in the present study, all experimental procedures were conducted to preserve uniformity and reproducibility. We believe that our results offer valuable information for orthopedists who perform pedicle screw instrumentation surgery.

## Conclusion

In healthy vertebrae, both conical and DC/DT designs can enhance the bone-screw interfacial strength. Among the six solid screws, the conical screw with the DC/DT profile presented the highest pullout strength (*p* < 0.05). A combination of the conical and DC/DT designs may achieve optimal postoperative screw stabilization. In osteoporotic bone, regardless of screw profile (SFT, SCT, or DC/DT), the strength was significantly higher with than without cement augmentation (*p* < 0.05). However, no significant difference was found among screws with the SFT, SCT, and DC/DT thread profiles (*p* > 0.05), suggesting that both the screw shape and thread profile have little impact on the screw fixation strength of pedicle screws fixed with cement in osteoporotic bone. Cement augmentation is the most important factor contributing to screw fixation strength in osteoporotic bone as compared to screw designs.

## Supporting information

S1 Table Maximal pullout strengths in six groups of synthetic vertebrae instrumented with solid screws. (PDF)

S2 Table Maximal pullout strengths in three groups of test blocks instrumented with cannulated cylindrical screws with and without cement augmentation. (PDF)
