# Supplementary material for: Biomechanical comparison of pedicle screw fixation strength in synthetic bones: Effects of screw shape, core/thread profile and cement augmentation
Source: PLoS One. 2020 Feb 21;15(2):e0229328. doi: 10.1371/journal.pone.0229328 (PMC7034823; doi:10.1371/journal.pone.0229328)
Supplement: S1 Table — (PDF) [file pone.0229328.s001.pdf]

Cylindrical screws

SFT	
Specimen No.	Max. Pullout Force (N)
1	457.15
2	662.13
3	512.97
4	421.22
5	647.49
6	521.37
Average	537.06
SD	98.46

SCT	
Specimen No.	Max. Pullout Force (N)
1	782.44
2	935.56
3	631.73
4	819.42
5	975.17
6	864.35
Average	834.78
SD	122.43

DC/DT	
Specimen No.	Max. Pullout Force (N)
1	1342.02
2	825.07
3	1013.51
4	979.35
5	921.11
6	1213.62
Average	1049.11
SD	192.65

Conical screws

SFT	
Specimen No.	Max. Pullout Force (N)
1	620.63
2	877.73
3	801.24
4	779.55
5	846.51
6	697.07
Average	770.46
SD	96.08

SCT	
Specimen No.	Max. Pullout Force (N)
1	931.79
2	893.37
3	921.12
4	789.68
5	917.47
6	785.59
Average	873.17
SD	67.45

DC/DT	
Specimen No.	Max. Pullout Force (N)
1	1452.98
2	1383.32
3	1078.66
4	1271.38
5	1362.7
6	1237.63
Average	1297.78
SD	132.63
