# Supplementary material for: Biomechanical comparison of pedicle screw fixation strength in synthetic bones: Effects of screw shape, core/thread profile and cement augmentation
Source: PLoS One. 2020 Feb 21;15(2):e0229328. doi: 10.1371/journal.pone.0229328 (PMC7034823; doi:10.1371/journal.pone.0229328)
Supplement: S2 Table — (PDF) [file pone.0229328.s002.pdf]

Cannulated screws with cement augmentation

SFT	
Specimen No.	Max. Pullout Force (N)
1	181.69
2	142.18
3	178.82
4	160.07
5	177.95
6	154.39
Average	165.85
SD	16.07

SCT	
Specimen No.	Max. Pullout Force (N)
1	198.56
2	142.77
3	198.52
4	169.17
5	149.83
6	184.41
Average	173.88
SD	24.06

DC/DT	
Specimen No.	Max. Pullout Force (N)
1	205.55
2	177.39
3	162.12
4	144.74
5	177.95
6	154.39
Average	170.36
SD	21.56

Cannulated screws without cement augmentation

SFT	
Specimen No.	Max. Pullout Force (N)
1	37.62
2	62.31
3	40.66
4	53.87
5	30.29
6	45.52
Average	45.05
SD	11.56

SCT	
Specimen No.	Max. Pullout Force (N)
1	104.82
2	113.99
3	91.71
4	86.33
5	74.5
6	79.08
Average	91.74
SD	15.18

DC/DT	
Specimen No.	Max. Pullout Force (N)
1	120.47
2	83.42
3	74.81
4	68.86
5	109.36
6	67.23
Average	87.36
SD	22.36
